# Differences in structural connectivity between diabetic and psychological erectile dysfunction revealed by network-based statistic: A diffusion tensor imaging study

**DOI:** 10.3389/fendo.2022.892563

**Published:** 2022-07-27

**Authors:** Jianhuai Chen, Jindan Wu, Xinfei Huang, Rui Sun, Ziliang Xiang, Yan Xu, Shi Chen, Weilong Xu, Jie Yang, Yun Chen

**Affiliations:** ^1^ Department of Andrology, Jiangsu Province Hospital of Chinese Medicine, Affiliated Hospital of Nanjing University of Chinese Medicine, Nanjing, China; ^2^ Department of Endocrinology, Nanjing First Hospital, Nanjing Medical University, Nanjing, China; ^3^ Department of Endocrinology, Jiangsu Province Hospital of Chinese Medicine, Affiliated Hospital of Nanjing University of Chinese Medicine, Nanjing, China; ^4^ Department of Urology, Jiangsu Provincial People’s Hospital, First Affiliated Hospital of Nanjing Medical University, Nanjing, China; ^5^ Department of Urology, People’s Hospital of Xinjiang Kizilsu Kirgiz Autonomous Prefecture, Artux, Xinjiang, China

**Keywords:** type 2 diabetes mellitus, erectile dysfunction, diffusion tensor imaging, network-based statistic, psychological erectile dysfunction

## Abstract

**Introduction:**

Type 2 diabetes mellitus (T2DM) has been found to be associated with abnormalities of the central and peripheral vascular nervous system, which were considered to be involved in the development of cognitive impairments and erectile dysfunction (ED). In addition, altered brain function and structure were identified in patients with ED, especially psychological ED (pED). However, the similarities and the differences of the central neural mechanisms underlying pED and T2DM with ED (DM-ED) remained unclear.

**Methods:**

Diffusion tensor imaging data were acquired from 30 T2DM, 32 ED, and 31 DM-ED patients and 47 healthy controls (HCs). Then, whole-brain structural networks were constructed, which were mapped by connectivity matrices (90 × 90) representing the white matter between 90 brain regions parcellated by the anatomical automatic labeling template. Finally, the method of network-based statistic (NBS) was applied to assess the group differences of the structural connectivity.

**Results:**

Our NBS analysis demonstrated three subnetworks with reduced structural connectivity in DM, pED, and DM-ED patients when compared to HCs, which were predominantly located in the prefrontal and subcortical areas. Compared with DM patients, DM-ED patients had an impaired subnetwork with increased structural connectivity, which were primarily located in the parietal regions. Compared with pED patients, an altered subnetwork with increased structural connectivity was identified in DM-ED patients, which were mainly located in the prefrontal and cingulate areas.

**Conclusion:**

These findings highlighted that the reduced structural connections in the prefrontal and subcortical areas were similar mechanisms to those associated with pED and DM-ED. However, different connectivity patterns were found between pED and DM-ED, and the increased connectivity in the frontal–parietal network might be due to the compensation mechanisms that were devoted to improving erectile function.

## Introduction

Type 2 diabetes mellitus (T2DM) is a chronic metabolic disorder characterized by hyperglycemia, insulin secretion dysfunction, and insulin resistance, which can lead to inflammation, oxidative stress, and endothelial dysfunction (1–3). T2DM has been identified to be associated with a variety of nervous system-related diseases and macro- and microvascular-related complications (4–6). The population-based studies suggested that the incidence of mild cognitive impairment in diabetic patients was around 21.8% in China and varied from 28 to 31.5% worldwide (7–9). An epidemiological study suggested that the prevalence of diabetes was increasing rapidly, and the prevalence of erectile dysfunction (ED) among diabetic patients varied from 35 to 90% (10). Compared with the general population, patients with T2DM have a higher risk for cognitive decline, which is one of the central nervous system complications associated with abnormalities of brain function and structure (11–13). In addition, T2DM patients are at higher risk of developing male sexual dysfunction, including ED and retrograde ejaculation, which are two common peripheral microvascular and neurological complications associated with oxidative stress-induced penile vascular endothelial cell injury and peripheral neuropathy (14–17).

ED is defined, in the Diagnostic and Statistical Manual of Mental Disorders (DSM-V) (18), as the inability to achieve and/or maintain an adequate erection until the completion of a sexual activity on 75% of attempts at a partnered sexual activity for satisfactory sexual intercourse or a marked decrease in turgidity ≥6 months with unsatisfactory sexual intercourse. Normal penile erection and detumescence is a complex neurovascular event that is regulated by the balance between the contraction and relaxation of cavernous smooth muscles (19). The etiological factors of ED can be classified as organic (neurogenic, arterial/venous, hormonal, and drug-induced) and psychological (20). Diabetes mellitus is considered as an important cause of organic ED (21, 22), while psychogenic ED (pED) is predominantly and exclusively attributed to psychological or interpersonal factors, such as performance anxiety and relationship stress (23). Hyperglycemia was considered to be associated with the development of impaired vasodilatory signaling, smooth muscle cell hypercontractility, and veno-occlusive disorder, which were all the mechanisms causing ED in T2DM patients and often led to resistance to current therapy (24, 25). Endothelial dysfunction was an important mechanism for the development of T2DM-related ED, and chronic hyperglycemia might lead to inflammation and contribute to the formation of reactive oxygen species, which were related to the development of endothelial dysfunction in T2DM-related ED (24). In addition, pED has been found to be related to impaired activity/functional connectivity and abnormal gray matter/white matter of the brain in recent functional and structural magnetic resonance imaging (MRI) studies (26–29). However, the neural mechanisms underlying T2DM, T2DM with ED (DM-ED), and pED remain unclear.

Diffusion tensor imaging (DTI) is a noninvasive MRI method that can be used to detect microstructural alterations of the white matter, which cannot be revealed by conventional structural MRI scan (30, 31). The integrity of nerve fibers can be measured by the parameter of fractional anisotropy (FA), which indicates the strength and direction of water molecules’ motion within the nerve fibers (32). Decreased FA values (values range from 0 to 1) indicate impaired microstructural tissue integrity of the white matter (33). A variety of white matter regions with microstructural alterations were found in T2DM patients by the technique of DTI (34). In addition, the structural brain networks [two elements: nodes defined by automated anatomical labeling (AAL) template; edges defined by white matter] of pED were constructed by the method of graph theory analysis, and the topological measures were compared with healthy controls (HCs) in our previous DTI study (26). The results showed that white matter fiber tracts connected with the left inferior frontal gyrus(triangular), amygdale, right inferior temporal gyrus, and rolandic operculum exhibited decreased strength of structural connectivity in pED patients, which was measured by FA value-weighted edges in the structural brain network (26).

Network-based statistic (NBS) is a validated nonparametrical statistical approach for elucidating the organization of brain while controlling family‐wise error. It is frequently applied to clinical applications, which can reveal altered connective strength in the brain network. To further identify different structural connections between pED and DM-ED, DTI data were acquired, and the approach of NBS was used in this study. We hypothesized that these patients would show a different structural connectivity located in key regions for sexual behavior regulation of the brain.

## Materials and methods

### Participants

In this cross-sectional study, a total of 93 patients, including 30 T2DM, 32 pED, and 31 DM-ED patients, were enrolled in this study. In addition, 47 age- and education-matched HCs were recruited by local advertisements. The protocol and informed consent document were approved by the Medical Ethics Committee of Jiangsu Province Hospital of Chinese Medicine, Affiliated Hospital of Nanjing University of Chinese Medicine. Written informed consents were obtained from all individuals before their participation in this study.

The inclusion criteria for all subjects were as follows: (1) right-handed, (2) educated for at least 9 years, and (3) aged between 20 and 60 years. The level of HbA1c was measured for the diagnosis of T2DM, and all participants were asked to fill in the five-item version of the international index of erectile function (IIEF-5) questionnaire to determine the presence of ED (35). T2DM patients met the diagnosis of T2DM according to the latest criteria published by the American Diabetes Association (ADA) (2014) (36): (1) fasting plasma glucose (FPG) level ≥7.0 mmol/L or (2) 2‐h oral glucose tolerance test glucose level ≥11.1 mmol/L. Patients with DM met the diagnosis of T2DM based on ADA criteria with IIEF-5 scores >21. Patients with pED met the diagnosis of ED based on DSM-V criteria with IIEF-5 scores ≤21 and normal erection during sleeping (normal morning erection) or masturbation (the penis could maintain an erection until ejaculation during masturbation) was reported by themselves as well as normal penile hemodynamics rated by the color duplex doppler ultrasonography combined with intracavernous injection. DM-ED patients met the diagnosis of T2DM (within 2 years) with the presence of ED (IIEF-5 scores ≤21; abnormal erection during sleeping and masturbation without obvious psychological factors, such as depression, anxiety, *etc.*). HCs were defined as individuals with normal FPG (<7.0 mmol/L), HbA1c (<6.5) level, and IIEF-5 scores >21.

The exclusion criteria for all individuals were as follows: (1) other types of diabetes, (2) history of severe hyperglycemia coma and hypoglycemia, (3) major medical illnesses or complications, such as severe liver, kidney, or cardiovascular disease or tumors, (4) psychiatric or neurologic disorders, (5) alcohol or other substance abuse, (6) organic brain lesions, such as brain injury, cerebrovascular lesions, or tumors, and (7) any MRI contraindication.

### MRI data acquisition

The MRI data were obtained with a 3.0-T MRI scanner (Siemens, Germany). All participants were instructed to relax with their eyes closed, keeping their heads still an avoid deliberate thinking and falling asleep during scanning. High-resolution sagittal three-dimensional T1-weighted images and DTI images were acquired with the parameters that have been described in our previous studies (37–40).

### Data preprocessing and network construction

**Figure 1:**
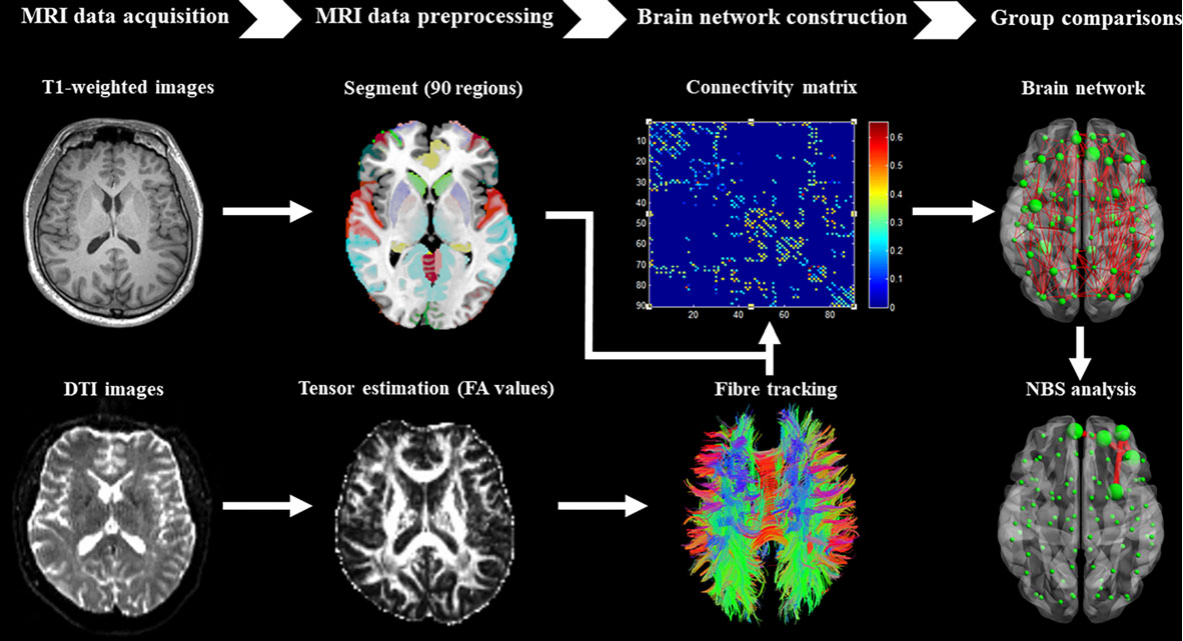
Brief flow chart showing MRI data acquisition, preprocessing, construction of structural brain network, and network-based statistical analysis between groups.

T1-weighted and DTI data were preprocessed using the diffusion toolbox of Functional MRI of the Brain software library (41). Then, whole-brain tractography was performed for the definition of edges in the brain network using the software of Diffusion Toolkit. In addition, the AAL template was used to define the nodes of the brain network (42). The detailed steps of preprocessing and construction of the whole-brain white matter network were performed (**Figure 1**) as reported in our previous studies (37–40).

### NBS analysis

NBS is a statistical method based on the graph theory and is often used to explore differences of the structural connectivity in the brain white matter network, which may be related to the diagnostic statue (43). NBS analysis is usually conducted to identify subnetworks comprising pairs of nodes and connections for which the strength of structural connectivity is significantly different between groups (44, 45). Firstly, two-sample *t*-tests were performed for all pairs (90 × 89/2 = 4,005) of nodes to test the null hypothesis of equality between groups in mean structural connectivity with respect to the size of interconnected subnetwork/component of edges rather than individually at each connection. Among the structural connections exceeding 2.5 (test–statistic threshold), the search was performed to identify any connected subnetwork, including a collection of regions and a set of suprathreshold connections. The size of the identified subnetwork was determined by the number of suprathreshold connections it comprised. Secondly, permutation tests were conducted to calculate the corrected *P*-value for each network. The size of the largest subnetwork was recorded, and the null distribution was generated for calculating the family-wise error-corrected statistical threshold across the set of all connections. Finally, the corrected *P*-value for the identified subnetwork (size = *K*) in the un-permuted/actual data was computed as the proportion of permutations for which the size of the subnetwork was equal or greater than *K*. Therefore, NBS is a statistical approach that controls the family-wise error rate across all connections of the brain network, which offers more power than the method of false discovery rate.

### Statistical analysis

The group differences of demographic and clinical variables were compared by using the SPSS software package (IBM, USA). The data normality was evaluated by Kolmogorov–Smirnov test, and the variance homogeneity was measured by Levene’s test. The one-way ANOVA was used to detect demographic and clinical differences among the three groups, while two sample *t*-test was performed to reveal differences of variables between two groups. The statistical significance threshold was set at *P* <0.05.

One-way ANOVA and *post-hoc* analysis with two-sample *t*-test were applied to identify the group differences of structural connectivity in the white matter brain network by the method of NBS. The connected subnetworks were considered to be significantly different if the corrected *P <*0.05 at the whole-network level with the preliminary statistic threshold 2.5 (50,000 permutations).

## Results

### Demographic and clinical characteristics

The demographic and clinical characteristics of the three groups are presented in **Table 1**. No significant differences were found in the age and educational level. Patients with pED and DM-ED had decreased IIEF-5 scores when compared to those with DM and HCs. In addition, there were no significant differences in the level of HbA1c between patients with DM and DM-ED.

**Table 1:** Demographic and clinical characteristics.

Variables	DM (*n* = 30)	pED (*n* = 32)	DM-ED (*n* = 31)	HCs (*n* = 47)	*F*/*t*	*P*
Age (years)	44.30 ± 8.03	42.69 ± 3.95	43.55 ± 9.82	43.19 ± 7.34	0.25	0.86
Education level (years)	14.80 ± 2.68	14.47 ± 2.51	14.48 ± 2.68	14.45 ± 1.60	0.17	0.92
IIEF-5 scores	23.53 ± 1.11	10.56 ± 5.07	15.23 ± 3.15	22.72 ± 0.68	153.63	<0.00
HbA1c (%)	8.24 ± 2.61	–	9.52 ± 2.52	–	-1.94	0.06

P <0.05 was considered to be statistically significant.

DM, diabetes mellitus; pED, psychological erectile dysfunction; DM-ED, diabetic erectile dysfunction; HCs, healthy controls; IIEF, international index of erectile function.

### Differences of structural connectivity revealed by NBS analysis

**Figure 2:**
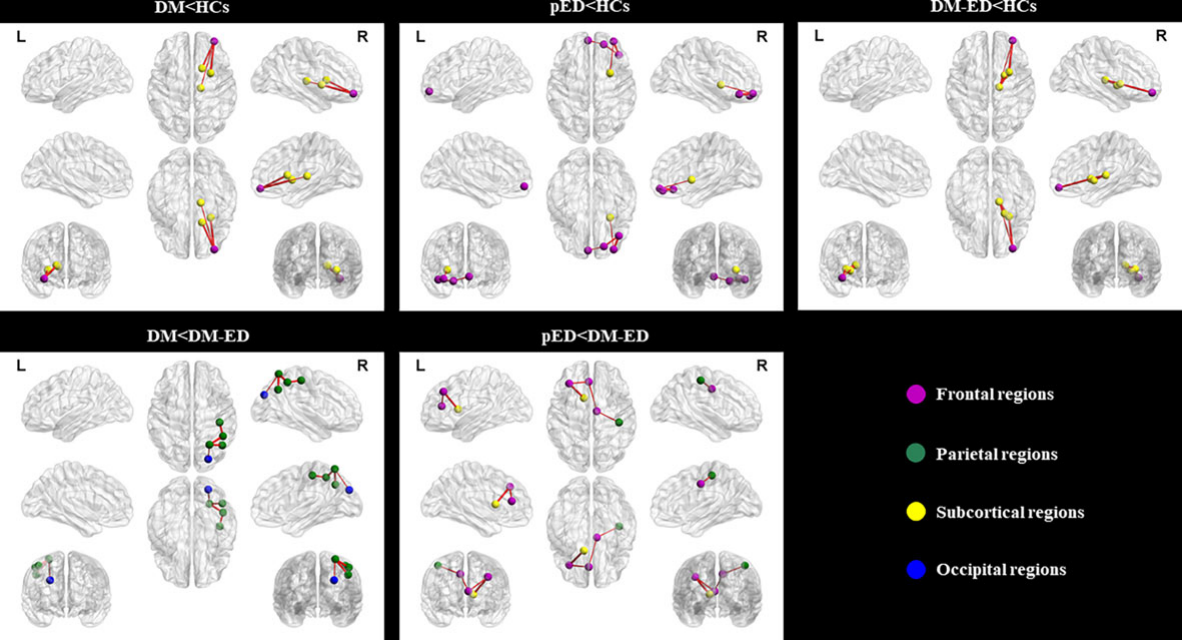
Subnetworks showing differences among the DM, pED, DM-ED, and HC groups using network-based statistical analysis. L, left; R, right; DM, diabetes mellitus; pED, psychogenic erectile dysfunction; DM-ED, diabetic erectile dysfunction; HCs, healthy controls.

As shown in **Table 2** and **Figure 2**, the subnetworks that showed significant differences between groups were identified. Compared to HCs, DM patients showed significantly decreased structural connectivity in a subnetwork comprising four brain regions (four right and zero left) and three connections (zero interhemispheric and three intrahemispheric). This subnetwork involved right middle frontal gyrus (orbital part), thalamus, putamen, and caudate nucleus. A subnetwork comprising five brain regions (four right and one left) and four reduced structural connections (one interhemispheric and three intrahemispheric) was identified in patients with pED when compared with HCs. In this subnetwork, the five well-connected brain regions were the left superior frontal gyrus (medial orbital) and right superior frontal gyrus (orbital part), inferior frontal gyrus (orbital part), middle frontal gyrus (orbital part), and putamen. The NBS analysis also revealed that a subnetwork was significantly different between DM-ED patients and HCs. The subnetwork consisted of four brain regions, including the right middle frontal gyrus (orbital part), thalamus, putamen, pallidum (four right and zero left) and four reduced structural connections (zero interhemispheric and four intrahemispheric).

**Table 2:** Subnetworks identified to be significantly different among the DM, pED, DM-ED, and HC groups using network-based statistical analysis.

Subnetwork	Edge		*t*	*P*
	Node 1	Node 2		
DM < HCs	Right middle frontal gyrus (orbital part)	Right thalamus	2.76	<0.05
	Right middle frontal gyrus (orbital part)	Right putamen	3.43	<0.05
	Right middle frontal gyrus (orbital part)	Right caudate nucleus	3.30	<0.05
DM > HCs	No significant edge was found			
pED < HCs	Left superior frontal gyrus (medial orbital)	Right superior frontal gyrus (orbital part)	2.53	<0.05
	Right superior frontal gyrus (orbital part)	Right inferior frontal gyrus (orbital part)	3.21	<0.05
	Right inferior frontal gyrus (orbital part)	Right middle frontal gyrus (orbital part)	4.22	<0.05
	Right middle frontal gyrus (orbital part)	Right putamen	2.52	<0.05
pED > HCs	No significant edge was found			
DM-ED < HCs	Right middle frontal gyrus (orbital part)	Right thalamus	2.81	<0.05
	Right middle frontal gyrus (orbital part)	Right putamen	3.78	<0.05
	Right thalamus	Right putamen	3.74	<0.05
	Right thalamus	Right pallidum	3.12	<0.05
DM-ED > HCs	No significant edge was found			
DM < DM-ED	Right superior parietal gyrus	Right inferior parietal gyrus	3.26	<0.05
	Right inferior parietal gyrus	Right postcentral gyrus	3.16	<0.05
	Right superior parietal gyrus	Right angular gyrus	3.18	<0.05
	Right superior parietal gyrus	Right superior occipital gyrus	2.91	<0.05
DM > DM-ED	No significant edge was found			
pED < DM-ED	Left middle frontal gyrus	Left caudate nucleus	4.21	<0.05
	Left middle frontal gyrus	Left anterior cingulate gyrus	3.16	<0.05
	Left anterior cingulate gyrus	Right median cingulate gyrus	2.70	<0.05
	Right median cingulate gyrus	Right postcentral gyrus	2.90	<0.05
pED > DM-ED	No significant edge was found			

To identify the significance of each subnetwork, nonparametric permutation statistic (test statistic threshold = 2.5; 5,000 permutations; P < 0.05) was performed with network-based statistical correction, and network size was measured with intensity.

DM, diabetes mellitus; pED, psychological erectile dysfunction; DM-ED, diabetic erectile dysfunction; HC, healthy controls.

In addition, the DM-ED patients demonstrated a subnetwork with five brain regions (five right and zero left) and four increased connections (zero interhemispheric and four intrahemispheric) when compared with DM patients. The regions of this subnetwork were located in the right superior parietal gyrus, inferior parietal gyrus, postcentral gyrus, angular gyrus, and superior occipital gyrus. Moreover, DM-ED patients had a different subnetwork comprising five brain regions (two right and three left) and four increased connections (one interhemispheric and three intrahemispheric) when compared with pED patients. The subnetwork consisted of the left middle frontal gyrus, anterior cingulate gyrus, caudate nucleus, right median cingulate gyrus, and postcentral gyrus.

## Discussion

To the best of our knowledge, this is the first study to explore the differences of structural connectivity between patients with pED and DM-ED by the method of NBS analysis. The findings demonstrated that decreased structural connectivity was found in patients with DM, pED, and DM-ED when compared with HCs. The abnormal brain regions were mainly distributed in the prefrontal and subcortical areas. In addition, DM-ED patients presented increased subnetworks consisting of parietal regions and prefrontal–cingulate areas when compared with DM patients and pED patients, respectively. These findings highlighted the importance of structural network analysis in understanding the different central neural mechanisms underlying diabetic and psychological ED.

In this study, we used DTI data to investigate the different topological properties of brain network between pED and DM-ED. Abnormal structural connectivity of white matter in the brain network were found in DM, pED, and DM-ED patients. The microstructural changes of white matter were speculated to be caused by the compromise of myelin sheath and the impairment or decrement of axons, which might lead to decreased neuronal signal transmission (34). The measure of FA, representing white matter integrity, is more sensitive than structural MRI metrics (33). DTI can detect and quantify subtle abnormalities of white matter before those are detectable by conventional structural MRI scans (30). Therefore, these findings might serve as imaging biomarkers for early diagnosis, monitoring disease progression, and response to therapy of brain disorders (46–48).

In recent years, DTI has been actively used in the investigation of brain structural connectivity alterations in sexual dysfunction patients including ED and premature ejaculation to understand the neuropathophysiology of these two disorders related to some psychological factors (26, 40). Our previous study showed that pED patients had damaged white matter in the left prefrontal and limbic cortex by the method of graph theoretical analysis (26). In addition, white matter microstructural changes were also found in pED patients by the method of tract-based spatial statistics based on DTI data (49). In this study, both pED and DM-ED patients showed lower structural connectivity in the prefrontal and subcortical areas when compared with HCs. Reduced structural connectivity was identified in the left superior frontal gyrus, right frontal regions, and putamen in pED patients, while DM-ED patients exhibited decreased structural connectivity in the right middle frontal gyrus, thalamus, putamen, and pallidum. This finding suggested that pED patients had more impairments in the frontal regions; however, DM-ED patients had more abnormalities in the subcortical areas. Previous studies demonstrated that the subcortical areas and, in particular, the thalamus seemed to be susceptible to T2DM. In addition, pED, owing predominantly to psychological factors including anxiety, depression, and introversion, was found to be more vulnerable to structural and functional changes in the prefrontal regions (26, 27, 38). Therefore, our findings were in agreement with the central neural mechanisms of pED and DM in previous neuroimaging studies (11, 38, 50).

The putamen was a key subcortical region receiving inputs from the prefrontal regions and projecting to other portions of the subcortical areas (51). The putamen, a critical component of the reward network, was considered to facilitate the integration of information from different brain areas and played an important role in reward-related behaviors (52). Sexual behavior was a subjectively pleasurable experience and activity which, in the putamen, could be triggered by visual sexual stimuli, which acted like rewarding stimuli (53–55). In previous neuroimaging studies, activation in the putamen was found to be associated with male sexual arousal and penile turgidity (56, 57). The interactions between the prefrontal and putamen were known to be important for reward and sexual behavior (58, 59). Impaired gray matter of the putamen was found in pED patients (60). The structural connectivity between the prefrontal and putamen might be abnormal and associated with the underlying neural mechanisms of pED. With the exception of the putamen, more subcortical regions, including thalamus and pallidum, were found to have reduced structural connectivity in DM-ED patients. The thalamus was considered as an integration center for different brain regions, and it was found to be a critical structure for cognitive dysfunction in T2DM patients (61, 62). Decreased FA value was found in the thalamus in diabetes mellitus patients when compared with HCs, and the decreased FA was associated with worse neurocognitive performance of patients (63). Both the putamen and pallidum were two important components of the striatum, which played a key role in various brain functions, including cognitive function and reward, through the cortico-striato-thalamo-cortical pathway (64–66). T2DM was often accompanied with ED, which might be also associated with the structural abnormalities in the brain as manifested by decreased structural connectivity in the striato-thalamo-frontal circuit.

In this study, increased structural connectivity was found in the frontal–parietal network of DM-ED when compared with DM and pED. The frontal–parietal network played a vital role in cognitive function, including attention, executive function, and working memory, and it was often activated by executive function-related tasks (67). In previous studies, the inferior parietal lobule was activated in response to visual sexual stimuli, and the regional cerebral blood flow of this region was found to be positively correlated with the level of penile tumescence (56, 68). The initiation and level of penile tumescence in response to visual sexual stimuli was controlled by the frontal–network (69). In addition, increased activation was found in the frontal–parietal network in youth with type 1 diabetes when compared with HCs (70). Therefore, the increased structural connectivity in the frontal–parietal network might indicate compensatory changes for DM-ED patients. However, the complex mechanisms underlying the compensatory changes needed to be explored in further studies with a larger sample size.

In addition, several limitations should be taken into consideration in this study. Firstly, the relatively small sample size and cross-sectional study might limit the generalizability of these findings. Secondly, more demographic and clinical characteristics should be obtained, and their relationships with altered structural connectivity in the brain network should also be explored in our future studies. Finally, future studies entailing longitudinal studies with treatment were needed to evaluate the alterations in brain structural connectivity under treatment and might provide new insight into the treatment strategy of ED.

## Conclusion

In summary, this might be the first study to investigate the differences of structural connectivity between diabetic and psychological ED by the method of NBS analysis based on DTI data. Our results showed that both DM-ED and pED had decreased structural connectivity in the frontal-subcortical regions. In addition, DM-ED patients presented increased structural connectivity in the frontal–parietal network, which might be a compensatory mechanism. These findings provided the first evidence of the common and different central neural mechanisms between diabetic and psychological factors related ED.

## Data availability statement

The raw data supporting the conclusions of this article will be made available by the authors, without undue reservation.

## Ethics statement

The studies involving human participants were reviewed and approved by the medical ethics committee of Jiangsu Province Hospital of Chinese Medicine, Affiliated Hospital of Nanjing University of Chinese Medicine. The patients/participants provided their written informed consent to participate in this study.

## Author contributions

JC, YC, JY, and JW designed the experiments. JC, JY, JW, XH, RS, ZX, YX, SC, and WX contributed to clinical data collection and assessment. JC, XH, ZX, YX, JW, and JY analyzed the results. JC, JY, JW, and XH wrote the manuscript. JC, YC, JY, and JW approved the final manuscript. All authors contributed to the article and approved the submitted version.

## Funding

This work was supported by grants from the National Natural Science Foundation of China (nos. 81701433 and 81871154), Key Project of Jiangsu Provincial Health Commission (no. ZDA2020025), Natural Science Foundation of Nanjing University of Chinese Medicine (no. XZR2020003), Special Project of Innovation and Development Fund of Jiangsu Province Hospital of Chinese Medicine (no. Y2021CX24), and General Project of Natural Science Foundation of Xinjiang Uygur Autonomous Region (2022D01A23).

## Conflict of interest

The authors declare that the research was conducted in the absence of any commercial or financial relationships that could be construed as a potential conflict of interest.

## Publisher’s note

All claims expressed in this article are solely those of the authors and do not necessarily represent those of their affiliated organizations, or those of the publisher, the editors and the reviewers. Any product that may be evaluated in this article, or claim that may be made by its manufacturer, is not guaranteed or endorsed by the publisher.
